# Coalescent: an open-source and scalable framework for exact calculations in coalescent theory

**DOI:** 10.1186/1471-2105-13-257

**Published:** 2012-10-03

**Authors:** Susanta Tewari, John L Spouge

**Affiliations:** 1 National Center for Biotechnology Information, Bethesda, MD, 20894, USA

**Keywords:** Population genetics, Object oriented design, Framework, Java, Netbeans platform, Coalescent, Recursion, Exact calculation

## Abstract

**Background:**

Currently, there is no open-source, cross-platform and scalable framework for coalescent analysis in population genetics. There is no scalable GUI based user application either. Such a framework and application would not only drive the creation of more complex and realistic models but also make them truly accessible.

**Results:**

As a first attempt, we built a framework and user application for the domain of exact calculations in coalescent analysis. The framework provides an API with the concepts of model, data, statistic, phylogeny, gene tree and recursion. Infinite-alleles and infinite-sites models are considered. It defines pluggable computations such as counting and listing all the ancestral configurations and genealogies and computing the exact probability of data. It can visualize a gene tree, trace and visualize the internals of the recursion algorithm for further improvement and attach dynamically a number of output processors. The user application defines jobs in a plug-in like manner so that they can be activated, deactivated, installed or uninstalled on demand. Multiple jobs can be run and their inputs edited. Job inputs are persisted across restarts and running jobs can be cancelled where applicable.

**Conclusions:**

Coalescent theory plays an increasingly important role in analysing molecular population genetic data. Models involved are mathematically difficult and computationally challenging. An open-source, scalable framework that lets users immediately take advantage of the progress made by others will enable exploration of yet more difficult and realistic models. As models become more complex and mathematically less tractable, the need for an integrated computational approach is obvious. Object oriented designs, though has upfront costs, are practical now and can provide such an integrated approach.

## Background

### Integrated approach

Current computational tools in population genetics do not follow an integrated approach. We define an integrated approach to be the one that allows reuse at the framework level and at the level of end user application, which allows running jobs developed by different researchers independently. Currently available applications in population genetics do not follow this approach. Each application is targeted towards a very specific use case where reuse and customization are a secondary issue. Most of these are targeted at native platforms and since coalescent computations can be intensive, optimizations are applied that tie the application further up with the platform. The problems this causes in developing and maintaining models in population genetics is well articulated by Felsenstein [1]. The primary reason for the lack of an integrated approach is probably the upfront costs involved. However, this need not be the case. In the past, probably it was impractical to follow the integrated approach but with the current abundance of cross-platform, open source technologies and the maturity of object oriented designs [2], it is very much practical. Success stories, such as the Netbeans platform [3], demonstrate that the costs involved in object oriented designs will be paid off tremendously with the reuse it allows. This is truer with coalescent analysis because the underlying theory naturally lends itself to object oriented design.

### First attempt: exact methods

With the maturity of the Java platform and the pace of open source development, it is practical to envision an object-oriented development in population genetics. Bioinformatics has already seen such efforts [4]. Recently, some efforts are underway in population genetics [5,6]. Exact methods, have recently gained in popularity [7-9], partly due to the increased computational power and partly for a need to evaluate available approximations. Nevertheless, exact methods are still not feasible for real data sets except for few cases [9]. Their primary value lies in evaluating approximate methods and gaining intuition to improve those approximate methods [10]. The authors have not seen any object oriented development for exact methods or a scalable GUI application in population genetics that allows running jobs in a plug-in like manner. The current paper describes such a first attempt.

### Overview of exact methods

An excellent overview of coalescent theory and applications is available from [11,12]. For the sake of completeness we briefly describe the models, the associated data and the calculations as implemented in the software. As a first attempt, we have considered only coalescent and mutation events in the model. Migration and recombination are the next important events to consider, but, we have omitted them for now as they introduce significant complexity into the models. We have considered the Infinite-Alleles Model (IAM) and the Infinite-Sites Model (ISM) as described in chapter 2.1 of [11]. IAM and ISM both postulate the creation of unique alleles on mutation. ISM is a sub-model of IAM that differs in the level of detail in the data. While, IAM data consists of frequencies of different alleles, ISM data additionally shows how the alleles are different by the site and the number of mutations.

### Phylogeny

**Figure 1:**
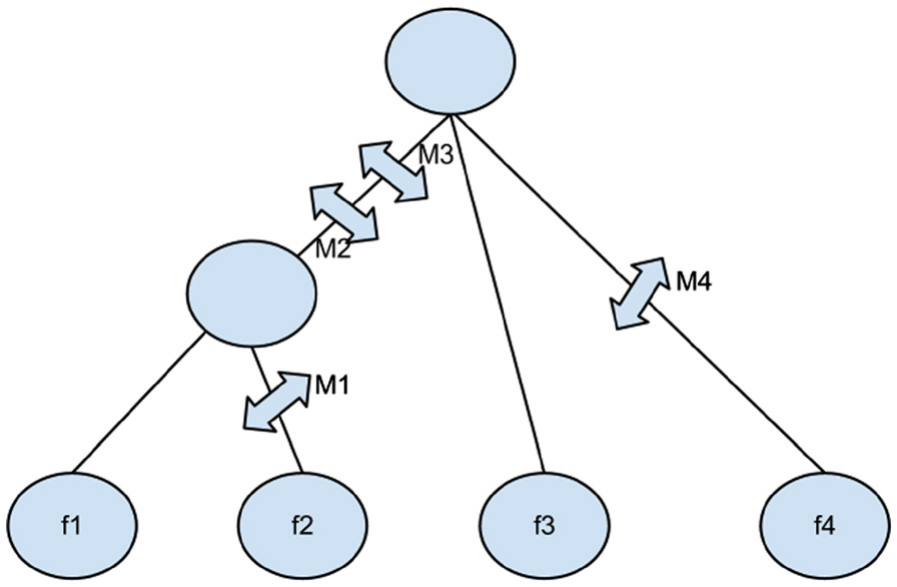
**Infinite**-**Sites Model Gene Tree.** Visualization of Equation 1 as a graph structure from the sample data to the most recent common ancestor using ancestral configurations and genealogies.

ISM data can be represented as a tree owing to the fact that each mutation is unique and creates a partition in the data. Such a tree is known as gene tree [Figure 1 in the literature. The corresponding binary data for the gene tree in [Figure 1 is given in [Table 1. A frequently useful computation is to check if a given ISM data set in the binary form actually constructs a gene tree or not. If it does, the data set is said to have the perfect phylogeny. There are two popular algorithms, Gusfield`s algorithm [13] and the Four Gametes [14] algorithm for checking perfect phylogeny. We have added a job (phylogeny/Check Phylogeny) in the application implementing this computation for both the algorithms. Another job (phylogeny/Visualize Gene Tree) creates a gene tree (as in [Figure 1) from a given ISM data in the binary form (as in [Table 1). This is useful because gene tree data structure clearly shows how mutations are shared among the alleles and is a more compact data structure for the ISM model than the corresponding binary form. 

**Table 1:** **Infinite**-**Sites Model Binary data**

**M1**	**M2**	**M3**	**M4**	**Frequency**
0	1	1	0	f1
1	1	1	0	f2
0	0	0	0	f3
0	0	0	1	f4

Data for Infinite-Sites model represented by 0s and 1s in a tabular form. Each column indicates a mutation site and each row an allele. The last column indicates the frequency of each allele.

### Recursion

Computing probability of a data set under a stochastic model is fundamental because it permits estimation of the model parameters using full information of the data. This should be contrasted with summary based statistics. Summary based statistics, though useful, are sub-optimal in nature. Except for few models (e.g., IAM), computing probability for most of the models in population genetics is non-trivial. They involve recursions which do not render themselves into analytical expressions. Recursions are common for almost all computations merely by the fact that data is correlated by the shared genealogies. The probability recursion for ISM is given by (see Equation 2.27 in [11]):

(1) $$\begin{array}{ll} n\left(n-1+\theta \right)P\left[T\right]= & \sum_{k\in S_{0}}n\left(n_{k}-1\right)P\left[C_{k}\left(T\right)\right]\\ & +\theta \sum_{k\in S_{1}}P\left[M_{k}\left(T\right)\right]\\ & +\theta \sum_{k\in S_{2}}\left(n_{i}+1\right)P\left[M_{k}\left(T\right)\right] \end{array}$$

where,

*S*_0_, *S*_1_ and *S*_2_ denote a partition of the alleles corresponding to the following events respectively: coalescent, mutation of the first kind and mutation of the second kind

If the removal of a mutation creates a unique allele in the data set then it is called a mutation of the first kind. The mutation of the second kind corresponds to a mutation whose removal creates an allele (called a *merge allele*) already present in the data set.

*i* denotes the merge allele for the corresponding mutation of the second kind

*C*_*k*_ denotes applying coalescent and *M*_*k*_ denotes applying mutation for allele *k* on gene tree*T*

*P*(.) is the probability function

*θ* is the population mutation rate

Probability of the most recent common ancestor is 1, which serves as the initial condition of the recursion

**Figure 2:**
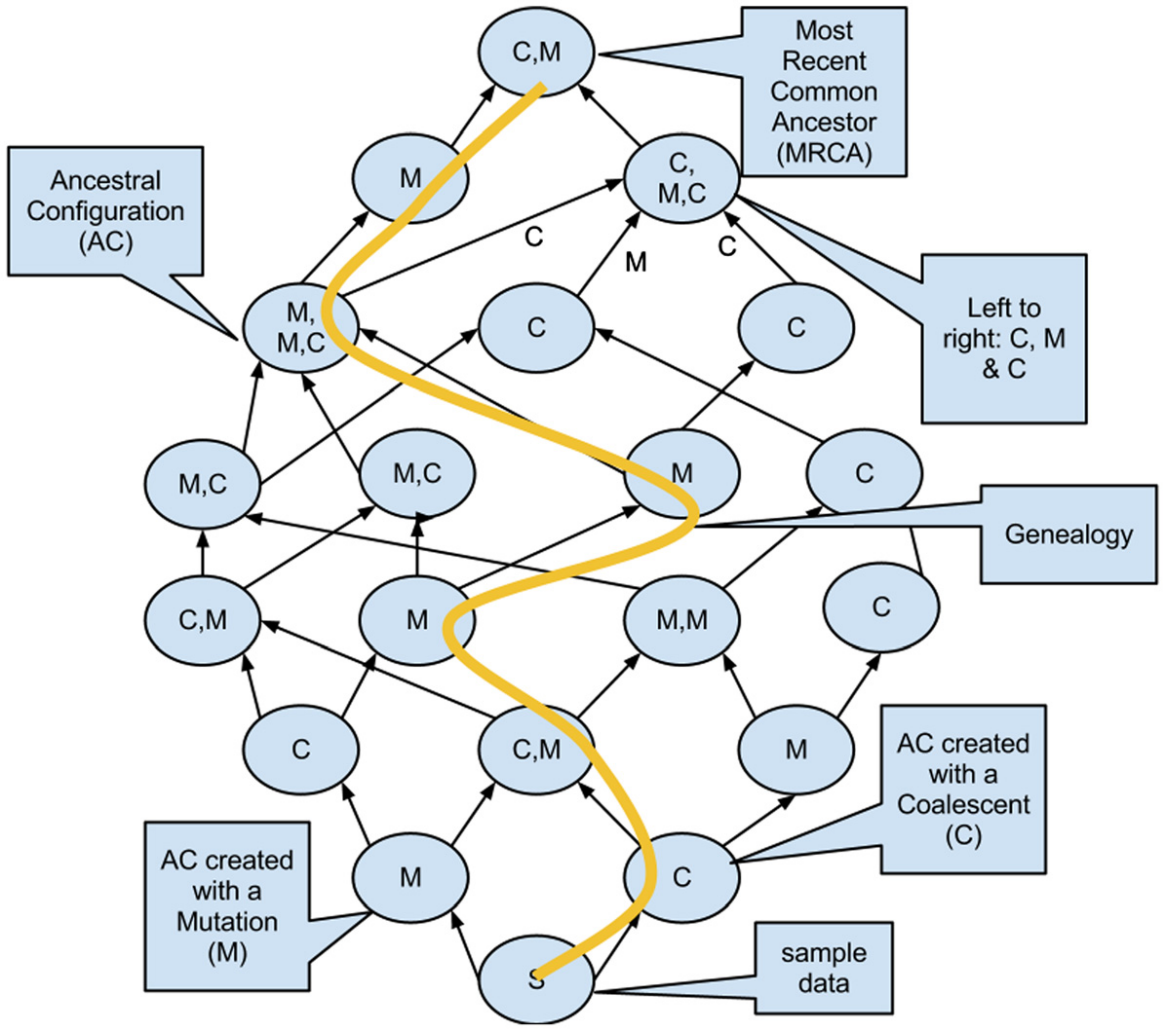
**Equation 1 Recursion Graph.** Visualization of Equation 1 as a graph structure from the sample data to the most recent common ancestor using ancestral configurations and genealogies.

The recursion graph represented by Equation 1 is shown in [Figure 2]. Solving Equation 1 requires understanding of the ancestral configurations and of the genealogies associated with the recursion graph. We have added options (*compute config count*, *build configs*) in the job ‘Recursion/Infinite-Sites Model’ to study the ancestral configurations. Other options (*compute genealogy count*, *build genealogies*) in the same job help us study the genealogies. Option (*compute exact probability*) to calculate the probability by solving Equation 1 is also present in the job.

## Implementation

### Core framework

The core framework models the concepts of population genetics and coalescent theory relevant for exact methods. It includes the concepts of model, data, statistic, phylogeny and recursion. The package *popgen*.*model* defines the infinite-alleles and infinite-sites model. Their corresponding data are defined in *popgen*.*data*. During the construction of infinite-sites data, it checks if the data conforms to the model assumption of phylogeny. Towards that end, the package, *popgen*.*phylogeny* defines two phylogeny algorithms- Gusfield`s algorithm [13] and the Four Gametes [14] algorithm. The package *popgen*.*statistic* contains statistics based on the data in *popgen*.*data*. For infinite-alleles data, the frequency spectrum sample configuration is defined as a statistic. For infinite-sites data, gene tree is defined as a statistic. The package *coalescent*.*recursion* forms the heart of exact computations by defining recursion in a generic way as a traversal (backward) of the sample configurations (*statistic*s) over the ancestral genealogies.

### Application framework

Part of the integrated approach is to be able to run jobs contributed by others. For example we have provided a GUI-based end-user application that is built on top of the Netbeans platform. This design should be contrasted with most of the available applications in population genetics, where the user interface and the application algorithms are intertwined in a manner that hinders reuse and extension. Besides making jobs reusable, another responsibility of the application framework is to provide common facilities to each job in a consistent manner from a central place. For example, attaching multiple output processors and algorithm profilers that collect useful data on the running algorithms, before or after the job has started, are part of this. These facilities have been implemented in the user application.

### Features

#### Phylogeny

##### Checks phylogeny of binary data

Running time is linear with data size. Large data sets will not take more than few seconds, if not instantaneous. The user has a choice between two popular algorithms, Gusfield`s algorithm and the Four Gamete`s algorithm. Gusfield`s algorithm is faster but Four Gamete`s algorithm is simpler to understand.

##### Draws phylogeny of binary data

For a given binary data set that has phylogeny (tested by the previous feature), it draws the corresponding gene tree. As discussed previously, data for infinite-sites model can be expressed both as arrays and tress. The gene tree representation has the distinct advantage of clearly visualizing which mutations are related to which alleles. Gusfield`s algorithm is used in creating this gene tree. The algorithm is fast enough that even large (~100 alleles) data sets would not take more than few seconds.

#### Recursion

A number of quantities related to the recursion (1) are computed. The size of the applicable data for these computations depends both on the number of mutations and the total number of alleles. A typical data set, about 35 alleles and around 10 mutations would not take more than 10 seconds on a PC with free RAM of 1GB. The available features on recursion are listed below.

##### Exact probability

The exact probability of the data and that of all its ancestral configurations are computed.

##### Counting ancestral configurations and genealogies

Total number of ancestral configurations and the total number of genealogies for a given data set are computed. These are important indicators of the complexity of the problem and are cited in the literature [7,9].

##### Builds ancestral configurations and genealogies

All the ancestral configurations and genealogies for a given data set are printed. Due to combinatorial nature of the problem, manual calculation of the ancestral configurations and that of the genealogies are extremely tedious even for small data sets (5 alleles and 4 mutations). It is important to note that intuitions on ancestral configurations and genealogies are critical in proposing better methods [10].

##### Profiles recursion cache

This is an advanced feature demonstrating how the entire framework can be used to improve on existing methods. This feature counts the number of ancestral configurations at each level of the recursion graph and plots the counts during the computation. This feature is currently being used by the authors to improve over the existing [9] algorithms for the traversal of the recursion graph.

## Results and discussion

**Figure 3:**
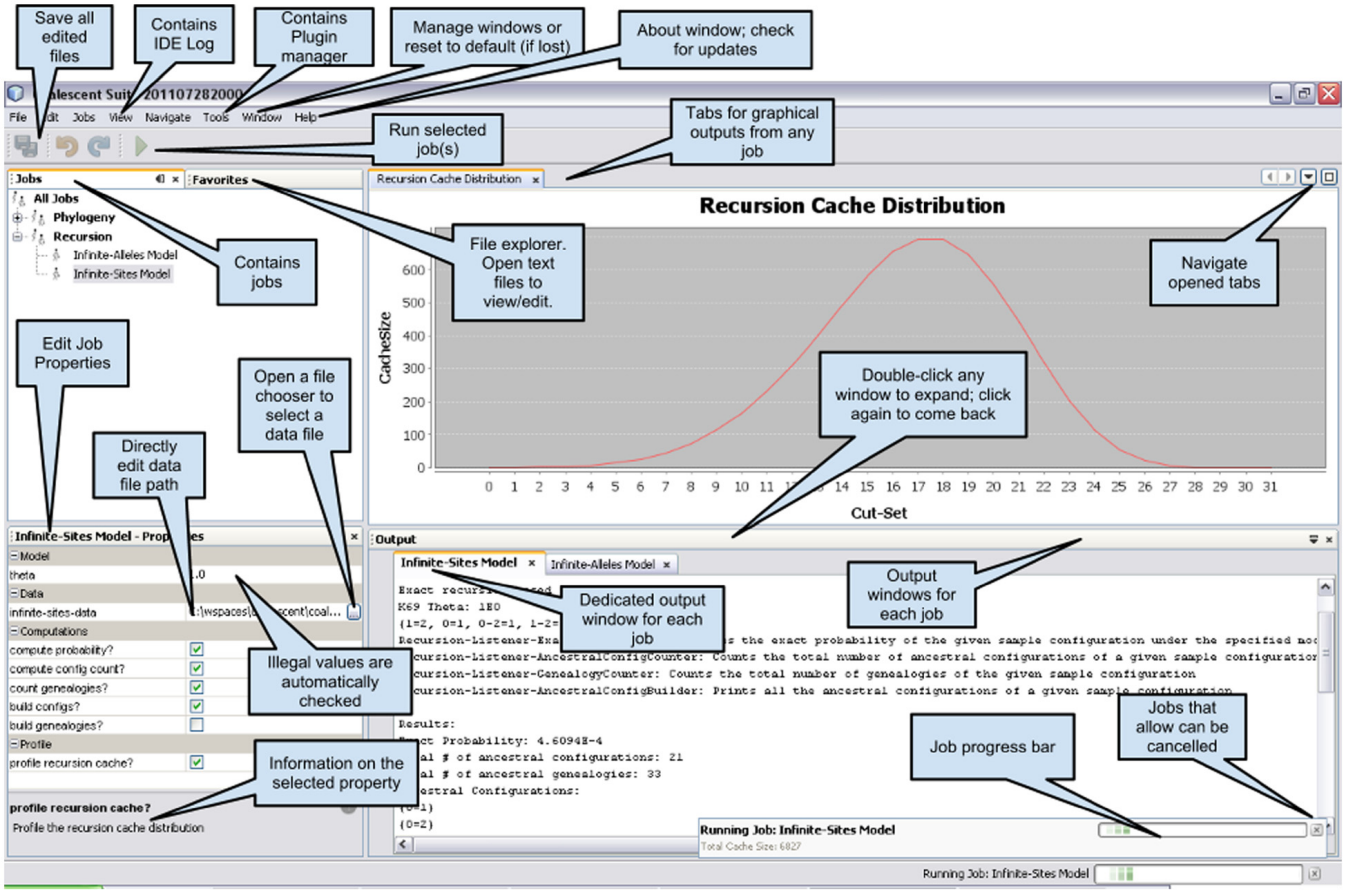
**Coalescent**-**1**.**3**.**0 running.** A Netbeans platform based end user GUI application, running jobs based on the core framework.

We have provided a core framework that has modelled some concepts of population genetics in an object oriented manner as required for exact methods. It has used design patterns to build a loosely coupled system that is extensible and scalable. It is coupled with an application framework that makes jobs reusable and provides the common facilities from a central place. An end user GUI application is built [see Figure 3 on top of the Netbeans platform that makes running, developing and sharing jobs easy. The framework and the end user application [see Additional file 1 and Additional file 2 along with the video tutorials on installing and running the application are available from the project website [15]. The development of this project will continue, particularly improving the recursion algorithm so that computations become feasible on bigger data sets and for models with migration and recombination. 

## Conclusions

With the current state of open source development, the maturity of operating system independent platforms and the object oriented designs, an integrated approach to computation is practical. An integrated approach allows exploration of more complex models by reuse. The reuse makes it practical to spend resources for continuous renovation. It has been the authors` observation that though there is upfront cost associated with the integrated approach, the benefits are worthwhile, because it promotes code reuse.

## Availability and requirements

**Project name**: coalescent

**Project home page**: http://coalescent.sourceforge.net

**Operating system(s)**: Platform independent

**Programming language**: Java

**Other requirements**: Java 1.7.0 or higher

**License**: GNU GPL v3

**Any restrictions to use by non-academics**: yes

## Competing interests

Both authors have no competing interest.

## Authors’ contributions

JLS conceived the project, motivated the GUI use cases, and provided feedback on tests. ST carried out the entire design and implementation. ST and JLS drafted the manuscript. Both authors read and approved the final manuscript.

## Supplementary Material

Additional file 1: Coalescent-1.3.0.zip (End user application) Description of Data: Unzip the file and go to the bin subdirectory. For windows run (double-click) the file coalescent.exe and for Mac/Linux run the file coalescent from the console. Running some jobs require infinite-sites data which is available as coalescent-1.3.0-data.zip. It contains the sample infinite-sites data files stored in xml format. A video tutorial on installation is available at http://youtu.be/f4Bye9jx81c?hd=1 and another on the application demo is available at http://youtu.be/IEuzpZZeV1Q?hd=1. Click here for file

Additional file 2: Coalescent-1.3.0-data.zip (Sample infinite-sites data) Description of data: Contains infinite-sites data stored in xml format. These files can be used while running jobs in the coalescent application. Click here for file
